# Comparative Lipidomics Analysis Provides New Insights into the Metabolic Basis of Color Formation in Green Cotton Fiber

**DOI:** 10.3390/plants13213063

**Published:** 2024-10-31

**Authors:** Tongtong Li, Congcong Zheng, Jianfei Wu, Wei Xu, Tongdi Yan, Junchen Liu, Li Zhang, Zhengmin Tang, Yupeng Fan, Huihui Guo, Fanchang Zeng

**Affiliations:** 1 State Key Laboratory of Crop Biology, College of Agronomy, Shandong Agricultural University, Tai’an 271018, China; lttsdau2019@163.com (T.L.); zccylxq@163.com (C.Z.); jfwu@sdau.edu.cn (J.W.); xwnxn2016@163.com (W.X.); m17861904530@163.com (T.Y.); gkoishi0514@gmail.com (J.L.); 15610418001@163.com (L.Z.); 17861500710@163.com (Z.T.); 2 College of Life Sciences, Huaibei Normal University, Huaibei 235026, China; fanyupeng@chnu.edu.cn

**Keywords:** colored cotton, fiber color formation, lipidomics, differential types of lipids, metabolic pathway

## Abstract

Green fiber (GF) is a naturally colored fiber. A limited understanding of its color formation mechanism restricts the improvement of colored cotton quality. This experiment used upland cotton green fiber germplasm 1-4560 and genetic inbred line TM-1; the lipid profiles of green fibers at 30 (white stage) and 35 days post-anthesis (DPA) (early greening stage), as well as those of TM-1 at the same stages, were revealed. Among the 109 differential types of lipids (DTLs) unique to GF, the content of phosphatidylserine PS (16:0_18:3) was significantly different at 30 and 35 DPA. It is speculated that this lipid is crucial for the pigment accumulation and color formation process of green fibers. The 197 DTLs unique to TM-1 may be involved in white fiber (WF) development. Among the shared DTLs in GF35 vs. GF30 and WF35 vs. WF30, sulfoquinovosyldiacyl-glycerol SQDG (18:1_18:1) displays a significant difference in the content change between green fibers and white fibers, potentially affecting color formation through changes in content. The enriched metabolic pathways in both comparison groups are relatively conserved. In the most significantly enriched glycerophospholipid metabolic pathway, 1-acyl-sn-glycero-3-phosphocholine (C04230) only appears in white cotton. This indicates differences in the metabolic pathways between white and green fibers, potentially related to different mechanisms of color formation and fiber development. These findings provide a new theoretical basis for studying cotton fiber development and offer important insights into the specific mechanism of green fiber color formation.

## 1. Introduction

Cotton is a crucial fiber crop globally. Naturally colored cottons (NCCs) eliminate the need for dyeing [1,2,3], thereby avoiding toxic pollutant treatment issues [4,5], with extensive developmental prospects in reducing production costs and safeguarding the environment. However, the pigment of colored cotton is unstable and monotonous, which severely limits the development of colored cotton [6].

NCCs primarily exhibit green and brown colors. Brown fiber pigments mainly consist of flavonoids [7,8,9] and proanthocyanidins [10,11,12]. Different flavonoids have varying impacts on fiber development. For instance, naringenin can significantly hinder fiber development [13]. *GhCHS*, a key gene in flavonoid biosynthesis, affects fiber color depth through its expression level [14]. Some researchers suggest that brown fiber pigments also contain catechins [15,16]. The composition of the green pigment is more complex than that of the brown color. Compared with white and brown cotton fibers, green cotton fibers have a different morphology, with a secondary cell wall of alternating cellulose–suberin layers [17]. Molecular analysis indicates that the pigment of green fibers is a caffeic acid derivative [18]. Caffeic acid is found in the suberin layer and alternates with cellulose outside the fiber [19]. Suberin is a polymer of aliphatic and aromatic domains [17]. Studies have identified cinnamic acid derivatives important for green fiber color formation, suberin coherence, and polymer attachment to cellulose cell walls. Glycerol is a new component of the green cotton fiber suberin layer [20].

Lipids play an important role in life activities, including energy storage, maintenance of the cell membrane structure, and signal transduction [21]. Transcriptome analysis of a green fiber cotton and its white fiber near-isogenic line revealed that differentially expressed genes are involved in lipid metabolism [22]. Green cotton fibers contain abundant waxes, with very long-chain fatty acids (VLCFAs) serving as precursors for wax synthesis [23]. Numerous genes related to long-chain fatty acids and VLCFAs were upregulated in the green cotton fibers. When shading treatment was applied to green fibers, they remained white instead of turning green. Metabolome analysis indicated that lipids constituted a significant proportion of differential metabolites [24].

Lipidomics, a branch of metabolomics, systematically analyzes complex lipid metabolic networks in living organisms [25]. Technological advances in mass spectrometry, chromatography, and Nuclear magnetic resonance (NMR) have driven the development of lipidomics. The main methods of lipidomics include nuclear magnetic resonance, shotgun, chromatography-mass spectrometry (MS), and liquid chromatography–MS (LC–MS) lipidomics. LC–MS/MS-based lipidomics is particularly effective in identifying low-abundance lipids.

The above studies provide a basis to explore the role of lipids in the growth of green fibers. In this study, non-targeted lipidomics analysis was conducted on the fibers of green cotton at the white stage and the early greening stage, as well as on the fibers of white cotton during the same period. This revealed the lipid composition in the green and white fibers at 30 and 35 days post-anthesis (DPA) and identified difference in lipid types between the green fiber (GF) and white fiber (WF). We identified differential types of lipids (DTLs) in GF associated with green color, providing a reference for analyzing the mechanism of green fiber color formation.

## 2. Results

### 2.1. Color Transformation During the Development of Green Fiber

**Figure 1:**
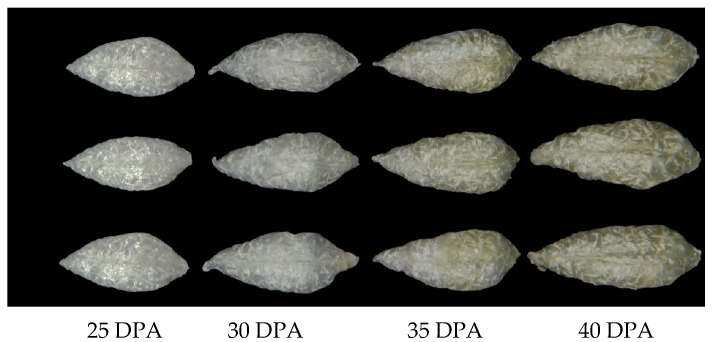
The color of fiber samples of upland cotton 1-4560 at different timepoints. From top to bottom, the samples range from 25 days post-anthesis (DPA) to 40 DPA.

The green fiber was white from 25 to 30 DPA, began to turn green at 35 days, and the color deepened over time (Figure 1). We measured three color parameters of upland cotton 1-4560 using a CR-300 Chroma Meter. As the fibers developed, the brightness (L*) of the green fibers gradually decreased. The a* values were negative and showed a decreasing trend, indicating that the green color deepened over time. The b* values were positive and gradually increased, representing a continuous deepening of the yellow color (Table 1).

### 2.2. Multivariate Statistical Analysis

**Figure 2:**
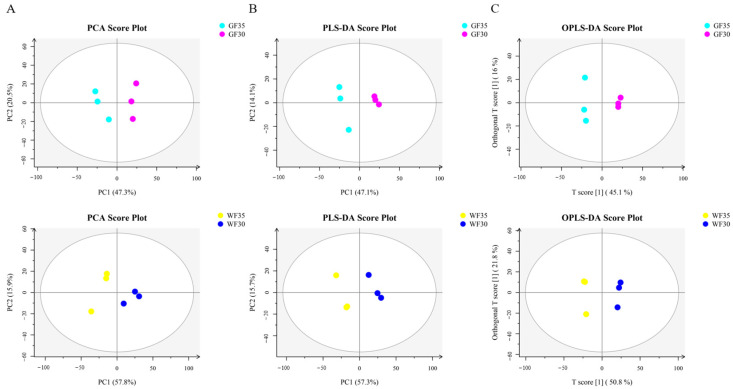
Multivariate statistical analysis. (**A**) Principal component analysis (PCA); (**B**) partial least squares-discriminant analysis (PLS-DA); (**C**) orthogonal partial least squares-discriminant analysis (OPLS-DA). GF30 represents the green fiber at 30 days post-anthesis; GF35 represents the green fiber at 35 days post-anthesis. WF30 represents the white fiber at 30 days post-anthesis; WF35 represents the white fiber at 35 days post-anthesis.

We conducted a multivariate statistical analysis on the lipids at two adjacent developmental stages of GF and WF (GF35 vs. GF30 and WF35 vs. WF30) to observe variability between the groups. Principal component analysis (PCA) modeling of the detected lipids revealed significant differences in lipid compositions between the groups (Figure 2A). The partial least squares-discriminant analysis (PLS-DA) (Figure 2B) and orthogonal partial least squares-discriminant analysis (OPLS-DA) (Figure 2C) score plots showed a clear group separation with high R^2^ and Q^2^ statistics (Table 2), indicating good fit and high predictability. These results suggest that the lipid dataset is robust and reliable for subsequent analysis.

### 2.3. Identification of DTLs and Venn Diagram Analysis

Suberin can also be classified as a lipid. However, since suberin is a polymer composed of aliphatic and aromatic domains, our experimental methods cannot directly detect the compositional changes in suberin. Therefore, our analysis examines lipids other than suberin. DTLs with a *p*-value ≤ 0.01 and a VIP ≥ 1 were considered significantly different. The statistical results of the DTLs are presented in Table 3.

In GF35 vs. GF30, 139 DTLs were identified, with 122 (87.8%) upregulated, including 4 AcHexChE, 4 AcHexCmE, 7 AcHexSiE, 6 AcHexStE, 4 AcHexZyE, 1 Cer, 1 ChE, 2 CmE, 10 DG, 4 DGDG, 1 LPE, 2 MG, 2 MGDG, 2 MePC, 6 PA, 11 PC, 9 PE, 6 PG, 1 PI, 1 PS, 1 SPH, 2 SQDG, 32 TG, 2 ZyE, and 1 PEt. Additionally, 17 (12.2%) DTLs were downregulated, including 1 BisMePA, 1 Cer, 1 CmE, 4 Hex1Cer, 1 MePC, 1 PC, 1 PI, 3 TG, 1 ZyE, 1 LPMe, 1 PMe, and 1 cPA (Table S1). In WF35 vs. WF30, 227 DTLs were identified, with 18 (7.9%) upregulated and 209 (92.1%) downregulated. Among the 18 upregulated lipids were 1 AcHexCmE, 7 Cer, 1 DG, 2 PA, 2 PC, 2 PE, 2 TG, and 1 Pet. Among the 209 downregulated lipids were 3 AcHexCmE, 1 AcHexStE, 4 AcHexZyE, 7 Cer, 1 CerP, 16 DG, 5 DGDG, 8 Hex1Cer, 1 MG, 2 MGDG, 14 MePC, 3 PC, 5 PE, 8 PG, 4 PI, 1 PS, 1 SPH, 2 SQDG, 1 ST, 94 TG, 2 WE, 1 ZyE, 2 DGDG, 1 LPC, 1 LPE, and 1 MGDG (Table S2). An analysis of the DTLs in these two comparison groups shows that most DTLs in GF35 vs. GF30 are upregulated, while most DTLs in WF35 vs. WF30 are downregulated. This indicates significant differences in lipid metabolism during fiber development and color formation in different types of cotton.

**Figure 3:**
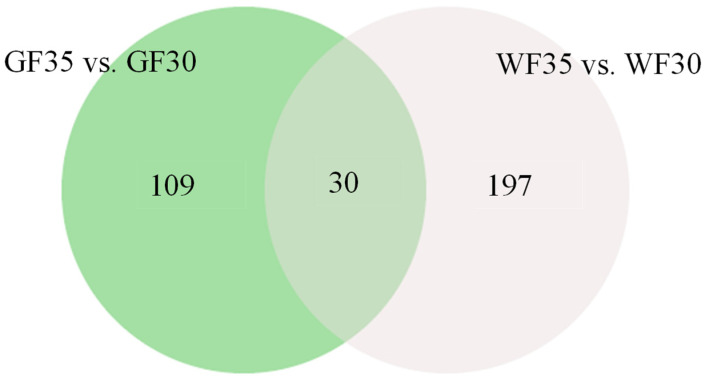
Venn diagrams of DTLs in the two comparison groups. Light green represents DTLs specific to GF35 vs. GF30, light gray represents DTLs specific to WF35 vs. WF30, and the overlapping section shows the 30 DTLs common to both comparison groups.

A total of 30 lipids showed significant content change in the GF and WF comparison group, indicating their important role in the development of both fiber types (Figure 3). These lipids include 3 ST, 3 SL, 3 SP, 8 GP, and 13 GL. Additionally, the fold change of SQDG (18:1_18:1) in WF35 vs. WF30 is much greater than in GF35 vs. GF30 (Table S3), suggesting its involvement in the differential phenotype formation of GF and WF and its potential role in regulating fiber color. Excluding these 30 shared lipids, 109 lipids are unique to green fibers, suggesting their role in green fiber pigment synthesis. Among them, PS (16:0_18:3) displays the highest fold change and is significantly different from other lipids. It can be regarded as a candidate lipid for regulating the color formation of green fibers. Subsequent experiments can further explore its specific functions and action mechanisms. For example, substances that promote or inhibit the synthesis of this lipid can be added to observe their impact on the development of green fibers. There were 197 DTLs unique to white fibers, indicating their association with white fiber development. We respectively screened out the top 10 specific DTLs that only appear in GF35 vs. GF30 and in WF35 vs. WF30 (Table 4). These included 1 SP, 1 ST, 8 GP, and 10 GL, suggesting a prominent role for GL in fiber development (Tables S4 and S5).

### 2.4. Components of DTLs in GF and WF at Different Developmental Stages

**Figure 4:**
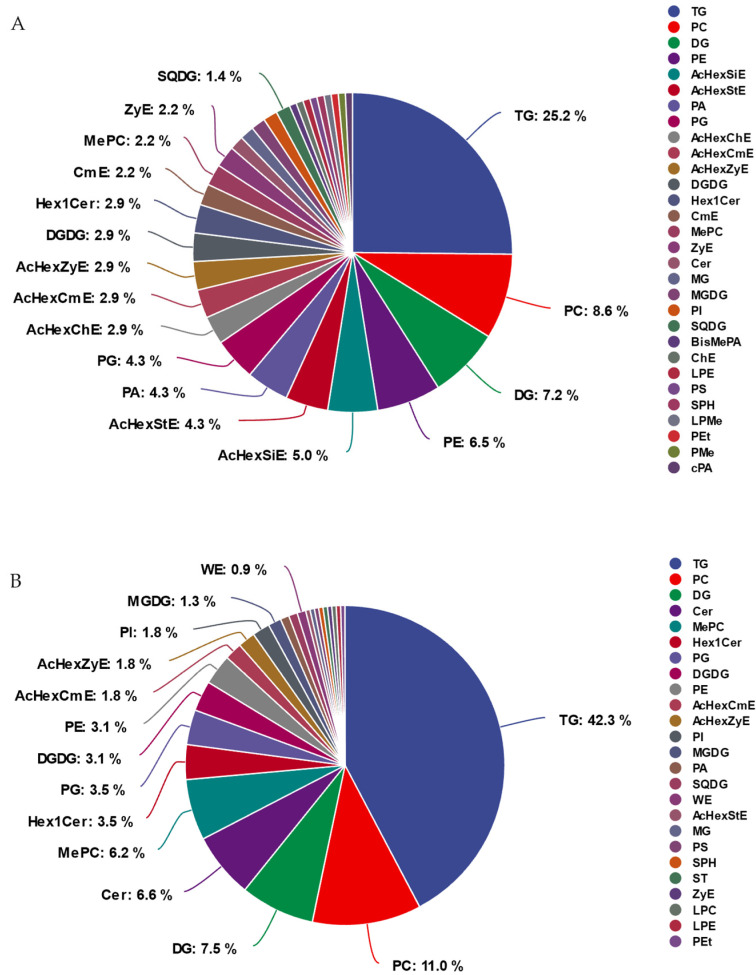
Component of the differential types of lipids (DTLs) in the two comparison groups. (**A**) DTLs in GF35 vs. GF30. (**B**) DTLs in WF35 vs. GF30. Each color represents a different lipid type. TG: triacylglycerol; PC: phosphatidylcholine; DG: diacylglycerol; PE: phospha-tidylethanolamine; AcHexSiE: acyl hexosyl sitosterol ester; AcHexStE: acyl hexosyl stigmasterol ester; PA: phosphatidic acids; PG: phosphatidylglycerols; AcHexChE: acyl hexosyl cholesterol ester; AcHexCmE: acyl hexosyl campesterol ester; AcHexZyE: acyl hexosyl zymosterol ester; DGDG: digalactosyl diacylglycerols; HexCer: hexosylceramide; CmE: cholesteryl methyl ester; MePC: methyl phosphatidylcholine; ZyE: zymosteryl; Cer: ceramides; MG: monoglyceride; MGDG: monogalactosyldiacylglycerol; PI: phosphatidylinositol; SQDG: sulfoquinovosyldiacyl-glycerol; BisMePA: phosphatidylmethanol; ChE: cholesteryl ester; LPE: lyso-phosphatidylethanolamine; PS: phosphatidylserine; SPH: sphingosine; LPMe: lyso-phosphatidylmethanol; PEt: phosphatidylethanol; PMe: phosphatidyl-methanol; cPA: cyclic phosphatidic acid; WE: wax ester; ST: sterol lipids; LPC: lyso-phosphatidylcholine.

To further explore lipid participation in GF and WF development, we conducted a classification analysis of DTLs in GF35 vs. GF30 and WF35 vs. WF30. Figure 4 shows that green cotton has more lipid types than white cotton (Figure 4). A total of 30 lipid subclasses are detected in the 139 DTLs of GF35 vs. GF30, while 25 lipid subclasses are detected in the 227 DTLs of WF35 vs. WF30. In GF and WF, TG, PC, and DG are all relatively large in proportion. TG displays the highest proportion, followed by PC, and DG ranks third. AcHexSiE, AcHexChE, CmE, BisMePA, ChE, PMe, and cPA only appear in green cotton, potentially playing specific physiological roles in the developmental process of green cotton fibers, while WE, ST, and LPC only appear in white cotton (Table S6). These differences indicate that green and white cotton exhibit distinct lipid metabolism patterns during fiber development.

### 2.5. Cluster and Volcano Plot Analysis of DTLs

**Figure 5:**
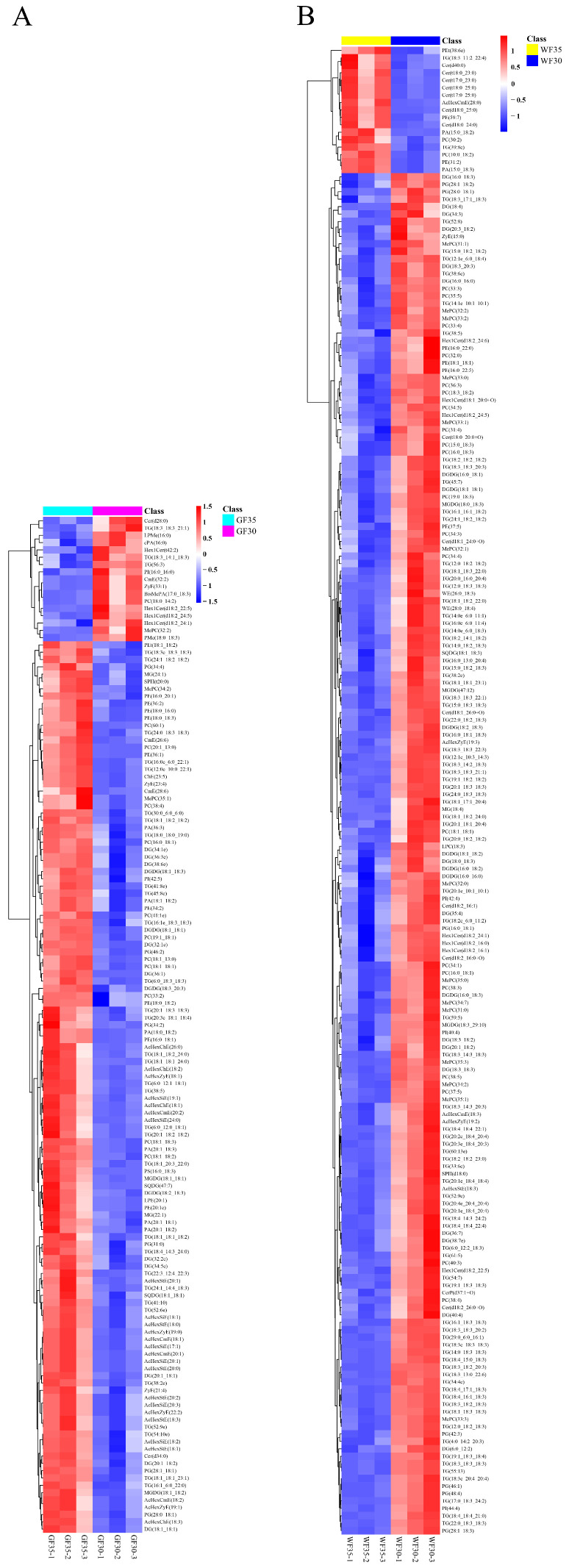
Clustering heat map analysis of DTLs. (**A**) A total of 139 upregulated or downregulated DTLs were visualized between GF35 and GF30. (**B**) A total of 227 upregulated or downregulated DTLs were visualized between WF35 and WF30. The red and blue colors in the graphs indicate upregulated and downregulated DTLs, respectively.

**Figure 6:**
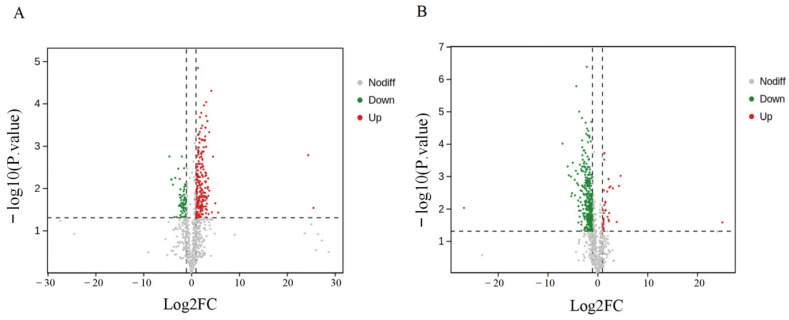
Volcano plot of DTLs. (**A**) DTLs upregulated or downregulated between GF35 and GF30. (**B**) DTLs upregulated or downregulated between WF35 and WF30. Each point in the volcano plot represents a lipid, with the *x*-axis representing the logarithm of the fold change of the lipid between the two samples and the *y*-axis representing the −log10 (*p* value). Red, blue, and gray colors in the figure indicate upregulated, downregulated, and not significantly different DTLs, respectively.

The clustering heat map (Figure 5) and volcano plot (Figure 6) intuitively show that the content change in the DTLs in the white cotton and green cotton comparison group is significantly different. In green cotton, most lipids are upregulated from 30 DPA to 35 DPA, while in white cotton, the opposite trend is observed. This indicates that lipids play a crucial role in fiber development and color formation in both cotton types.

**Figure 7:**
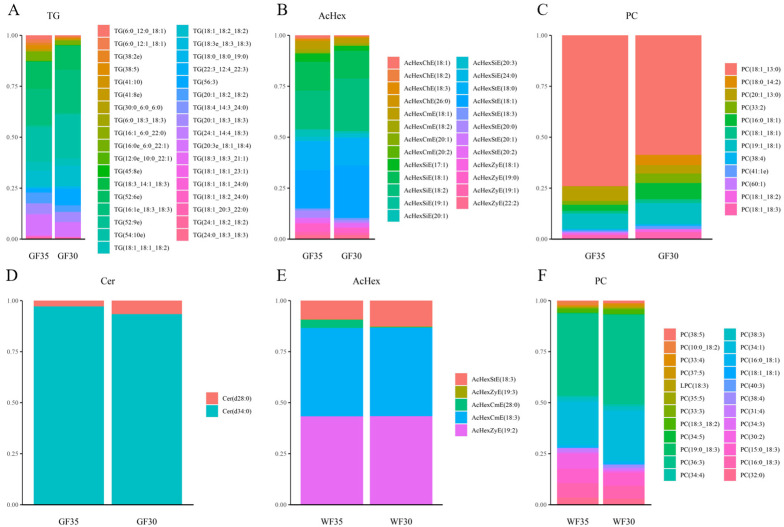
Typical differential lipid categories. (**A**) Distribution of TG lipids in GF35 vs. GF30; (**B**) distribution of AcHex lipids in GF35 vs. GF30; (**C**) distribution of PC lipids in GF35 vs. GF30; (**D**) distribution of Cer lipids in GF35 vs. GF30; (**E**) distribution of AcHex lipids in WF35 vs. WF30; (**F**) distribution of PC lipids in WF35 vs. WF30. TG: triacylglycerol; AcHex: including AcHexChE (acyl hexosyl cholesterol ester), AcHexCmE (acyl hexosyl campesterol ester), AcHexSiE (acyl hexosyl sitosterol ester), AcHexStE (acyl hexosyl stigmasterol ester), and AcHexZyE (acyl hexosyl zymosterol ester); PC: phosphatidylcholine; Cer: ceramides. The modules of different colors represent different lipids.

In GF35 vs. GF30, the triglyceride (TG) subclass had the most DTLs (35), indicating its high activity from 30 to 35 DPA in green cotton (Figure 7A). The AcHex subclass showed a large difference in lipid quantity between green and white cotton. In GF35 vs. GF30, 25 AcHex DTLs showed a significant content change, compared to only 5 in the WF35 vs. WF30 group (Figure 7B,E). Conversely, the PC subclass had fewer DTLs in the GF35 vs. GF30 compared to the WF35 vs. WF30 group (Figure 7C,F); additionally, two Cer subclass lipids showed content change only in the GF35 vs. GF30 group (Figure 7D).

### 2.6. Correlation Analysis of DTLs

**Figure 8:**
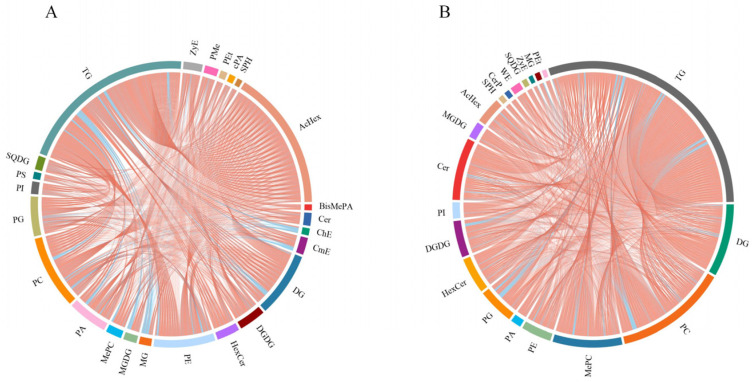
Correlation chord diagram of DTLs. (**A**) Correlation between DTLs in GF35 vs. GF30; (**B**) correlation between DTLs in WF35 vs. WF30. Each color around the circle represents a different lipid type, and the line color inside the circle represents the correlation between two lipids. Orange lines represent positive correlation, and blue lines represent negative correlation. TG: triacylglycerol; SQDG: sulfoquinovosyldiacylglycerol; PS: phosphatidylserine; PI: phosphatidylinositol; PG: phosphatidylglycerols; PC: phosphatidylcholine; PA: phosphatidic acids; MePC: methyl phosphatidylcholine; MGDG: monogalactosyldiacylglycerol; MG: monoglyceride; PE: phosphatidylethanolamine; HexCer: hexosylceramide; DGDG: digalactosyl diacylglycerols; DG: diacylglycerol; CmE: cholesteryl methyl ester; ChE: cholesteryl ester; Cer: ceramides; BisMePA: bis-methyl phosphatidic acid; AcHex: including AcHexChE (acyl hexosyl cholesterol ester), AcHexCmE (acyl hexosyl campesterol ester), AcHexSiE (acyl hexosyl sitosterol ester), AcHexStE (acyl hexosyl stigmasterol ester), and AcHexZyE (acyl hexosyl zymosterol ester); SPH: sphingosine; cPA: cyclic phosphatidic acid; PEt: phosphatidylethanol; PMe: phosphatidyl methanol; ZyE: zymosteryl; CerP: ceramides phosphate; WE: wax ester.

The correlation analysis of DTLs can reveal the trend of changes between different lipids. Lipids showing similar trends are positively correlated, while those showing opposite trends are negatively correlated. Most of the DTLs in GF35 and GF30 show similar trends, indicating a positive correlation in content change between these lipids (Figure 8A). At the same time, the DTLs in WF35 and WF30 show the same trend (Figure 8B). These results indicate that although there are many unique lipids involved in the developmental processes of green cotton and white cotton, the changing trends among these lipids are relatively conservative. This further proves the regulatory role of lipids in this process.

### 2.7. Lipid Metabolism Pathway of Fibers

**Figure 9:**
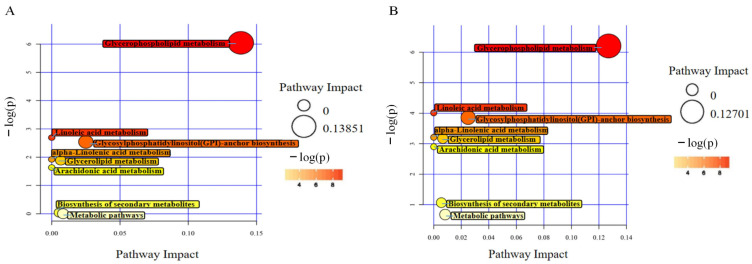
Bubble chart of KEGG influencing factors of DTLs. (**A**) KEGG pathways enriched in DTLs of GF35 vs. GF30; (**B**) KEGG pathways enriched in DTLs of WF35 vs. WF30. The *x*-axis represents the impact value of the enrichment in different metabolic pathways, and the *y*-axis represents the −log(*p*) value. Each point represents a metabolic pathway. The larger the impact value, the larger the dot, indicating a greater influence of that metabolic pathway. The color is related to the −log(*p*) value; the larger the −log(*p*) value, the deeper the color.

To elucidate the metabolic pathways involved in fiber development, the DTLs of the GF and WF comparison groups were enriched, respectively, using the Kyoto Encyclopedia of Genes and Genomes (KEGG) database. The analysis revealed fewer enriched pathways due to the lack of corresponding lipid identifiers in KEGG. The significantly enriched metabolic pathways in both comparison groups are shown in Figure 9, including the glycerophospholipid metabolism, glycosylphosphatidylinositol (GPI) anchor biosynthesis, linoleic acid metabolism, glycerolipid metabolism, α-linolenic acid metabolism, and arachidonic acid metabolism pathways. In the components of these pathways, phosphatidylethanolamine (C00350), phosphatidylcholine (C00157), and triacylglycerol (C00422) appear in both green and white cotton. However, in the most significantly enriched glycerophospholipid metabolism pathway, 1-acyl-sn-glycero-3-phosphocholine (C04230) only appears in white cotton. This indicates differences in the fiber development between white and green cotton. Glycerophospholipids are an important component of cell membranes. Their metabolism may affect the growth, differentiation, and function of fibers. Further research on this pathway’s specific mechanisms could provide new ideas and methods for promoting fiber development. These findings provide an important entry point for further research regarding the differences between WF and GF and help to provide a deeper understand of the complex mechanism of fiber color formation and the unique metabolic characteristics of different fiber types during their development.

## 3. Discussion

Lipids are a class of metabolites. They constitute the main component of the biological membrane and regulate signal transduction processes such as cell growth, differentiation, senescence, and programmed cell death. At the same time, lipids can also provide energy, participate in the growth of organisms, and maintain life activities [26]. Lipids are the basic substances that organisms use to perform many crucial functions. Therefore, lipid metabolism is a major research hotspot in biological metabolism [27]. In terms of maintaining the normal physiological functions of cells, lipids are as important as genes and proteins [28]. As a key branch field of metabolomics, lipidomics can permit a comprehensive and in-depth systematic analysis of the entire set of lipids in specific cells or organisms, enabling a more thorough understanding of the complex lipid metabolism network and also revealing the core and crucial functions that lipids play in life activities.

### 3.1. Lipid Profiles of WF and GF

Previous studies have pointed out that the growth and development of fibers depend on the synthesis and transportation of long-chain fatty acids [29,30]. In this study, we investigated the lipid changes of GF and WF at 30 and 35 DPA. The results showed that there were 139 DTLs in GF35 vs. GF30 and 227 DTLs in WF35 vs. WF30. Among them, 30 lipids show significant content changes in both GF35 vs. GF30 and WF35 vs. WF30. They mainly belong to two subclasses, glycolipids (GL) and glycerophospholipids (GP). Notably, the changes in these 30 lipids in green fibers and white fibers showed different upregulation and downregulation patterns. This phenomenon further indicates that the differential change patterns of lipids reflect their potentially crucial influence on the fiber development process, and there are differences in the utilization and regulation mechanisms of lipids between green and white cotton.

### 3.2. Conserved Lipid Metabolic Pathways with Differential Components in GF and WF

DTLs are enriched in multiple identical pathways, including glycerophospholipid metabolism, GPI anchor biosynthesis, linoleic acid metabolism, glycerolipid metabolism, α-linolenic acid metabolism, and arachidonic acid metabolism pathways, in the developmental comparison groups of green and white cotton. This means that these metabolic pathways may play important and relatively stable roles in the development of different types of cotton fibers. In the components of the glycerophospholipid metabolism pathway, 1-acyl-sn-glycero-3-phosphocholine (C04230) only appears in white cotton. The presence of C04230 may be related to the specific physiological process of white cotton. Follow-up studies can further explore key differentially expressed genes in these pathways. Then, whether or not they affect the formation or accumulation of fiber pigments can be further verified, thus providing important clues and a basis for an in-depth understanding of cotton fiber development and pigment synthesis mechanisms.

### 3.3. Effect of Lipid on Color Formation of Green Cotton

Green fibers are unstable and easily decolorized by sunlight and rain [31]. Genes related to metal transporter proteins are highly expressed in green cotton, with higher contents of Fe^2+^ and Cu^2+^ than those noted in white fibers. These metals may chelate with pigment substances, altering fiber color [32]. Metabolomic and transcriptomic analyses reveal differences in the pathways related to the biosynthesis of phenylpropanoids, cutin, suberin, and wax between green and white cotton. The relationship between these factors and the formation of green fiber color requires further exploration [22].

Green and white cotton fibers show distinct differences in the aliphatic components of suberin polymers. The C_22_ chain length is dominant (95.5%) in the polymer monomers of green fibers, while the aliphatic polymers of ordinary white cotton fibers are mainly composed of dihydroxyhexadecanoic acid. Cotton fiber wax primarily comprises wax esters and fatty alcohols. In green fiber, wax esters release predominantly C_22_ (54%) fatty acids, while in white cotton, they mainly release C_16_ (26%) [17]. Studies in Arabidopsis thaliana also found that lipids play a role in plant color formation. After transforming *antisense acyl carrier protein-4*, lipid synthesis in leaf tissue is reduced, and the leaves turn white [33]. To further explore the color formation mechanism of green fibers, we analyzed the DTLs unique to green fibers. PS (16:0_18:3) shows a significant content change during the developmental stage from 30 DPA to 35 DPA and only occurs in green cotton. Phosphatidylserine (PS), an essential constituent of eukaryotic membranes, is the most abundant anionic phospholipid in the eukaryotic cells, accounting for up to 10% of the total cellular lipids, playing an important role in cell signal transduction [34]. Therefore, we speculate that PS (16:0_18:3) may play a role in the mechanism regulating fiber color. In addition, among the shared DTLs in GF35 vs. GF30 and WF35 vs. WF30, SQDG (18:1_18:1) displays a different change pattern in the two comparison groups. Therefore, SQDG (18:1_18:1) may affect fiber color through different levels of lipid content. Further experiments can be conducted to verify whether PS (16:0_18:3) and SQDG (18:1_18:1) are involved in the color formation of green fibers and to analyze the specific mechanism employed.

## 4. Materials and Methods

### 4.1. Plant Materials and Growth Conditions

Upland cotton 1-4560 (green fiber) and the genetic standard line TM-1 (white fiber) were ordered from the germplasm center of the Institute of Cotton Research, CAAS. These were planted in the experimental field of Shandong Agricultural University, with alternating wide (80 cm) and narrow (60 cm) row spacing and a 28 cm spacing between plants. Flowers from the first and second fruiting nodes on the middle and upper branches of the main stem were self-pollinated and tagged at full bloom. Three cotton bolls of similar size from green cotton (1-4560) and white cotton (TM-1) at 30 DPA and 35 DPA were harvested. They were stripped in the field, transported to the laboratory in liquid nitrogen, and stored at −80 °C for lipidomic analysis. Each group consisted of three biological replicates.

### 4.2. Determination of Green Fiber Color

Fiber color was determined using a Minolta CR-300 Chroma Meter (Osaka, Japan). A CIE light source was selected, and a white calibration plate was used for calibration before the assay. CIE L*a*b* was used to indicate green cotton fibers’ color and dynamic changes during cotton fiber development [35]. Assay color values were captured in the L*a*b* color space. The L* value (0 means black, and 100 means white) denotes the sample brightness. The a* value indicates the color density from red to green, with positive values indicating red and negative values indicating green. The b* value indicates the color density from blue to yellow, with positive values indicating yellow and negative values indicating blue. The coordinate origin represents the colorless colored dots [36]. The L*, a*, and b* color values were measured five times for each period.

### 4.3. Lipid Extraction

The lipid extraction procedure was as follows: First, 100 mg of each fiber sample was placed into a 2 mL centrifuge tube. Then, 750 μL of a precooled (−20 °C) chloroform–methanol solution (2:1, *v*/*v*) and two steel balls were added. The samples were ground using a high-flux tissue grinder at 60 Hz for 60 s. After grinding, the samples were placed on ice for 40 min. Next, 190 μL of ddH_2_O was added, and the mixture was vortexed thoroughly for 30 s before being placed on ice for an additional 10 min. The mixture was then centrifuged at 12,000 rpm for 5 min at room temperature. Subsequently, 300 μL of the lower phase was transferred to a new 2 mL centrifuge tube. An additional 500 μL of the precooled (−20 °C) chloroform–methanol solution (2:1, *v*/*v*) was added, vortexed thoroughly for 30 s, and centrifuged again at 12,000 rpm for 5 min at room temperature. After centrifugation, 400 μL of the lower phase was transferred to a new 2 mL centrifuge tube. The sample was then concentrated using a vacuum centrifuge concentrator. The concentrated sample was dissolved in 200 μL of isopropanol and filtered through a 0.22 μm membrane to obtain the lipid sample for high-performance liquid chromatography–mass spectrometry (LC–MS) analysis. Finally, 20 μL of each sample was pooled to create a quality-control (QC) sample [37,38].

### 4.4. The Chromatographic Conditions

The chromatographic separation was carried out using a Thermo Ultimate 3000 system with an ACQUITY UPLC^®^ BEH C18 column (100 × 2.1 mm, 1.7 µm, Waters, Milford, MA, USA) maintained at 50 °C. The autosampler temperature was maintained at 8 °C. Analytes were eluted with a gradient of acetonitrile/water (60:40, 0.1% formic acid + 10 mM ammonium formate) (C) and isopropanol/acetonitrile (90:10, 0.1% formic acid + 10 mM ammonium formate) (D) at 0.25 mL/min. Each sample (2 μL) was injected after equilibration. The linear gradient of solvent C (*v*/*v*) increased as follows: 0~5 min, 70~57% C; 5~5.1 min, 57~50% C; 5.1~14 min, 50~30% C; 14~14.1 min, 30% C; 14.1~21 min, 30~1% C; 21~24 min, 1% C; 24~24.1 min, 1~70% C; 24.1~28 min, 70% C.

### 4.5. Mass Spectrometry Conditions

ESI-MSn experiments were executed on a Thermo Q Exactive Focus mass spectrometer (Thermo Fisher Scientific, Waltham, MA, USA), with the spray voltage of 3.5 kV (positive mode) and −2.5 kV (negative mode). The sheath gas and auxiliary gas were set at 30 and 10 arbitrary units, respectively, with a capillary temperature of 325 °C. The Orbitrap analyzer scanned a mass-to-charge ratio (*m*/*z*) range of 150–2000 at a resolution of 35,000. Data-dependent acquisition (DDA) MS/MS experiments were conducted using an HCD scan with a normalized collision energy of 30 eV. Dynamic exclusion was applied to eliminate redundant information in the MS/MS spectra [39].

### 4.6. Data Processing

Lipidsearch software (V4) was used to annotate the raw data, generating a data matrix including M/Z, retention time (RT), and intensity. The annotation results of all samples were aligned and filtered using Lipidsearch software (v4.0). The main parameters were set to an r.t. tolerance of 0.25 and an m-score threshold of 5 [40]. To compare data of different magnitudes, the total peak intensity was normalized, and lipids were classified by molecular structure and hydrophobicity [41].

### 4.7. Statistical Analysis

The lipid data were processed using Pareto scaling, followed by multivariate statistical analysis including principal component analysis (PCA), partial least squares-discriminant analysis (PLS-DA), and orthogonal partial least squares-discriminant analysis (OPLS-DA) using the R language ropls package [42]. KEGG is a comprehensive database integrating information on genomes, biological pathways, diseases, drugs, and chemicals [43]. Lipid sequences were aligned to the KEGG database using Blast software (BLAST+ 2.13.0) [44] to obtain KO numbers and extract the associated KEGG pathways.

## 5. Conclusions

In this study, non-targeted lipidomics were used to reveal the lipid profiles of GF and WF at 30 and 35 DPA. We identified 1184 lipids in four fiber samples. By analyzing the DTLs in GF35 vs. GF30 and WF35 vs. WF30, the metabolic pathways in which lipids are involved for fiber development and color formation were identified. Among them, the glycerophospholipid metabolism pathway is the most significant. Among the DTLs in the two developmental comparison groups, we found that PS (16:0_18:3) and SQDG (18:1_18:1) display significantly different content change patterns in GF and WF, suggesting that they are involved in the color formation of green fibers. This experiment is conducive to an in-depth understanding of the molecular mechanism of fiber development and color formation and provides valuable scientific support for cotton variety improvement and fiber quality enhancement. In the future, it is expected that it will be possible to change the color of cotton fibers by regulating the expression of key genes regulating the synthesis of pigments, suberin, and lipids.

## Figures and Tables

**Table 1:** Dynamic changes in color values of green fiber during development.

Cultivars	Color Value	Days Past Anthesis (D)					
		25 D	30 D	35 D	40 D	45 D	50 D
Upland cotton 1-4560	L*	90.35	89.90	90.19	83.13	75.65	68.74
	a*	−0.61	−1.43	−2.31	−2.55	−2.48	−4.87
	b*	5.89	7.79	9.50	12.53	16.55	16.91

**Table 2:** Model parameters of GF35 vs. GF30 and WF35 vs. WF30 for comparative analysis.

Group	Type	PRE	R^2^X	R^2^Y	Q^2^
GF35 vs. GF30	PCA	2	0.678		
	PLS-DA	2	0.612	0.997	0.89
	OPLS-DA	2	0.61	0.997	0.892
WF35 vs. WF30	PCA	2	0.737		
	PLS-DA	2	0.73	0.996	0.95
	OPLS-DA	2	0.727	0.996	0.923

**Table 3:** Statistics of DTLs.

Simple	Control	Upregulated Lipids	Downregulated Lipids	Differential Types of Lipids
GF35	GF30	122	17	139
WF35	WF30	18	209	227

**Table 4:** Specific DTLs among the groups.

Group	Lipid Name	Category	Log2 (Fold Change)
Specific DTLs (GF35 vs. GF30)	PS (16:0_18:3)	GP	24.44
	PC (60:1)	GP	4.55
	PMe (18:0_18:3)	GP	−4.55
	DG (36:1)	GL	4.20
	BisMePA (17:0_18:3)	GP	−4.14
	PC (18:0_14:2)	GP	−4.13
	TG (6:0_18:3_18:3)	GL	3.76
	TG (18:1_18:1_24:0)	GL	3.54
	TG (6:0_12:0_18:1)	GL	3.49
	PC (20:1_13:0)	GP	3.38
Specific DTLs (WF35 vs. WF30)	TG (18:3_14:2_18:3)	GL	−5.97
	TG (52:8)	GL	−5.74
	TG (18:3_14:3_18:3)	GL	−5.30
	PC (40:3)	GP	−5.25
	DG (18:4)	GL	−5.10
	TG (17:0_18:3_24:2)	GL	−5.00
	Cer (d18:0_25:0)	SP	4.64
	TG (54:7)	GL	−4.56
	WE (26:0_18:3)	ST	−4.50
	PI (44:4)	GP	−4.32

Log2 (fold change), with two decimal places reserved.

## Data Availability

The data are contained within the article.

## Supplementary Materials

The following supporting information can be downloaded at: https://www.mdpi.com/article/10.3390/plants13213063/s1, Table S1: DTLs of GF35 vs. GF30; Table S2: DTLs of WF35 vs. WF30; Table S3: Shared DTLs of GF35 vs. GF30 and WF35 vs. WF30; Table S4: Specific DTLs in GF35 vs. GF30; Table S5: Specific DTLs in WF35 vs. WF30; Table S6: Statistics of lipid species in GF35 vs. GF30 and WF35 vs. WF30.
