# Supplementary material for: Comparative Lipidomics Analysis Provides New Insights into the Metabolic Basis of Color Formation in Green Cotton Fiber
Source: Plants (Basel). 2024 Oct 31;13(21):3063. doi: 10.3390/plants13213063 (PMC11548578; doi:10.3390/plants13213063)
Supplement: Supplementary file 1 [file plants-13-03063-s001.zip › plants-3281654-supplementary.pdf]

# Supplementary data:

**Table S1.** DTLs of GF35 vs. GF30.

| Lipid name | log2 (fold change) | VIP | Lipid name | log2 (fold change) | VIP |
|---|---|---|---|---|---|
| AcHexChE (18:1) | 2.929293746 | 1.412428425 | PE (16:0_18:1) | 1.727510812 | 1.461013517 |
| AcHexChE (18:2) | 2.306939018 | 1.406505523 | PE (34:2) | 1.389193722 | 1.416662114 |
| AcHexChE (18:3) | 1.973972434 | 1.42171146 | PE (36:1) | 3.379417819 | 1.473085899 |
| AcHexChE (26:0) | 1.672123195 | 1.384675514 | PE (36:2) | 2.012488261 | 1.422288607 |
| AcHexCmE (18:1) | 2.106338679 | 1.444500115 | PE (18:0_18:2) | 1.279176752 | 1.373651766 |
| AcHexCmE (18:2) | 1.785615993 | 1.378709945 | PE (18:0_18:3) | 1.553400106 | 1.447274861 |
| AcHexCmE (20:1) | 2.772845796 | 1.459035905 | PG (31:0) | 1.15972898 | 1.40493168 |
| AcHexCmE (20:2) | 2.513425274 | 1.424688844 | PG (28:0_18:1) | 1.290725208 | 1.409017764 |
| AcHexSiE (17:1) | 2.106338679 | 1.444500115 | PG (46:2) | 3.130810233 | 1.480976441 |
| AcHexSiE (18:1) | 1.435231092 | 1.448757358 | PG (28:1_18:1) | 1.739006786 | 1.457262731 |
| AcHexSiE (18:2) | 0.915980281 | 1.391092513 | PI (16:0_16:0) | -2.392720567 | 1.396531992 |
| AcHexSiE (19:1) | 2.267477082 | 1.423548898 | PI (42:5) | 1.036789596 | 1.385348767 |
| AcHexSiE (20:1) | 3.089213989 | 1.453052586 | PS (16:0_18:3) | 24.44397528 | 1.442777032 |
| AcHexSiE (20:3) | 1.4325449 | 1.401752591 | SPH (t20:0) | 0.548792425 | 1.401834772 |
| AcHexSiE (24:0) | 1.625337497 | 1.389697203 | SQDG (18:1_18:1) | 1.769392225 | 1.40168432 |
| AcHexStE (18:0) | 1.435874558 | 1.4489775 | TG (6:0_12:0_18:1) | 3.486609184 | 1.373133844 |
| AcHexStE (18:1) | 0.918542538 | 1.389675743 | TG (6:0_12:1_18:1) | 3.113871718 | 1.405769263 |
| AcHexStE (18:3) | 1.294712311 | 1.392598074 | TG (38:2e) | 2.86669018 | 1.459852218 |
| AcHexStE (20:0) | 3.089213989 | 1.453052586 | TG (38:5) | 3.11292087 | 1.410765577 |
| AcHexStE (20:1) | 0.978030687 | 1.418228977 | TG (41:10) | 2.29948597 | 1.447326976 |
| AcHexStE (20:2) | 1.43198254 | 1.401737293 | TG (41:8e) | 2.322743681 | 1.437536705 |
| AcHexZyE (18:1) | 2.240610353 | 1.406462237 | TG (30:0_6:0_6:0) | 2.089087858 | 1.46881114 |
| AcHexZyE (19:0) | 2.111697294 | 1.445731278 | TG (6:0_18:3_18:3) | 3.76078143 | 1.466062429 |
| AcHexZyE (19:1) | 1.785906063 | 1.378619056 | TG (16:1_6:0_22:0) | 0.673076547 | 1.393560509 |
| AcHexZyE (22:2) | 1.432567407 | 1.401689463 | TG (16:0e_6:0_22:1) | 2.404737792 | 1.459988794 |
| BisMePA (17:0_18:3) | -4.135506018 | 1.395752146 | TG (12:0e_10:0_22:1) | 2.332368662 | 1.470475901 |
| Cer (d28:0) | -0.838639384 | 1.369782856 | TG (45:8e) | 1.826999985 | 1.390163657 |
| Cer (d34:0) | 0.429374876 | 1.433074912 | TG (18:3_14:1_18:3) | -1.069300666 | 1.376335322 |
| ChE (23:5) | 2.960747644 | 1.469166762 | TG (52:6e) | 1.439230325 | 1.452092343 |
| CmE (26:6) | 2.132279882 | 1.440958684 | TG (16:1e_18:3_18:3) | 1.010648953 | 1.421510066 |
| CmE (28:6) | 2.938231699 | 1.369905663 | TG (52:9e) | 1.348675018 | 1.394410351 |
| CmE (32:2) | -0.246981341 | 1.399283363 | TG (54:10e) | 0.93512999 | 1.388880674 |
| DG (32:1e) | 1.393135096 | 1.488103225 | TG (18:1_18:1_18:2) | 1.515571037 | 1.387239281 |
| DG (32:2e) | 1.071705863 | 1.424997889 | TG (18:1_18:2_18:2) | 0.926462018 | 1.462614704 |
| DG (34:1e) | 1.790203226 | 1.439420944 | TG (18:3e_18:3_18:3) | 1.399180054 | 1.42913567 |
| DG (34:5e) | 1.064437216 | 1.417443601 | TG (18:0_18:0_19:0) | 0.195486842 | 1.412127473 |
| DG (36:1) | 4.195572129 | 1.484105588 | TG (22:3_12:4_22:3) | 1.500244209 | 1.443740133 |
| DG (18:1_18:1) | 2.379611695 | 1.424703888 | TG (56:3) | -0.841021517 | 1.420536302 |

| | | | | | |
|---|---|---|---|---|---|
| DG (36:3e) | 1.251644428 | 1.403310384 | TG (20:1_18:2_18:2) | 1.987238332 | 1.378736079 |
| DG (20:1_18:1) | 2.592117239 | 1.447469599 | TG (18:4_14:3_24:0) | 2.014092512 | 1.412066364 |
| DG (20:1_18:2) | 1.555148927 | 1.433674824 | TG (20:1_18:3_18:3) | 1.300615312 | 1.406870296 |
| DG (38:6e) | 1.251644428 | 1.403310384 | TG (24:1_14:4_18:3) | 1.846227807 | 1.429412631 |
| DGDG (18:1_18:1) | 2.684110335 | 1.479890486 | TG (20:3e_18:1_18:4) | 1.816291096 | 1.401233358 |
| DGDG (18:1_18:3) | 1.035624905 | 1.423331213 | TG (18:3_18:3_21:1) | -1.428130179 | 1.373437123 |
| Hex1Cer (d18:2_22:5) | -3.33887471 | 1.399337896 | TG (18:1_18:1_23:1) | 1.434685511 | 1.446099199 |
| Hex1Cer (d18:2_24:1) | -0.848278825 | 1.383111194 | TG (18:1_18:1_24:0) | 3.53817035 | 1.398008078 |
| Hex1Cer (d18:2_24:5) | -2.689054856 | 1.420252911 | TG (18:1_18:2_24:0) | 1.882359295 | 1.370228221 |
| Hex1Cer (t42:2) | -1.481763662 | 1.42113076 | TG (18:1_20:3_22:0) | 2.255089561 | 1.459765295 |
| LPE (20:1) | 3.062762427 | 1.389602362 | TG (24:1_18:2_18:2) | 1.411340081 | 1.463909571 |
| MG (22:1) | 1.247416759 | 1.400957832 | TG (24:0_18:3_18:3) | 1.503262746 | 1.446672736 |
| MG (24:1) | 1.526718316 | 1.403013578 | ZyE (21:4) | 1.341482067 | 1.376398715 |
| MGDG (18:1_18:2) | 2.421385835 | 1.40201156 | ZyE (23:4) | 2.960091338 | 1.469308208 |
| MePC (32:2) | -3.805227939 | 1.380230555 | ZyE (33:1) | -0.246981341 | 1.399283363 |
| MePC (34:2) | 1.314596961 | 1.424033197 | DGDG (18:2_18:3) | 1.038156213 | 1.395811333 |
| MePC (35:1) | 2.265084044 | 1.381939618 | DGDG (18:3_20:3) | 0.73901626 | 1.456778286 |
| PA (18:0_18:2) | 1.727510812 | 1.461013517 | LPMe (16:0) | -1.962904773 | 1.440842953 |
| PA (18:1_18:2) | 1.389193722 | 1.416662114 | MGDG (18:1_18:1) | 2.35444369 | 1.436073034 |
| PA (36:3) | 1.201807091 | 1.441078478 | PA (20:1_18:1) | 2.307714965 | 1.428825271 |
| PC (18:1_13:0) | 2.923978171 | 1.462839214 | PA (20:1_18:2) | 2.561542727 | 1.418717252 |
| PC (18:0_14:2) | -4.133017738 | 1.394777267 | PA (20:1_18:3) | 3.118814674 | 1.475551839 |
| PC (20:1_13:0) | 3.379417819 | 1.473085899 | PC (18:1_18:2) | 1.826848076 | 1.474962759 |
| PC (33:2) | 1.292993922 | 1.377021196 | PC (18:1_18:3) | 1.799283165 | 1.466334862 |
| PC (16:0_18:1) | 1.195313934 | 1.404764482 | PE (16:0_20:1) | 1.587232297 | 1.464701413 |
| PC (18:1_18:1) | 2.157580428 | 1.460020122 | PEt (18:1_18:2) | 2.587103324 | 1.452468423 |
| PC (19:1_18:1) | 2.105400699 | 1.476974485 | PG (34:2) | 2.902785868 | 1.38981104 |
| PC (38:4) | 2.269306842 | 1.383434036 | PG (34:4) | 1.633404345 | 1.429123496 |
| PC (41:1e) | 1.572289816 | 1.45216311 | PMe (18:0_18:3) | -4.555008203 | 1.440889212 |
| PC (60:1) | 4.554500477 | 1.440669748 | SQDG (47:7) | 2.934827468 | 1.404038052 |
| PE (20:1e) | 3.032696692 | 1.387594126 | cPA (16:0) | -1.303898228 | 1.380870422 |
| PE (18:0_16:0) | 1.553400106 | 1.447274861 | | | |

**Table S2.** DTLs of WF35 vs. WF30.

| Lipid name | log2 (fold change) | VIP | Lipid name | log2 (fold change) | VIP |
|---|---|---|---|---|---|
| AcHexCmE (18:2) | -1.375817716 | 1.296589513 | ST (t18:1_24:0+O) | -2.139959266 | 1.297388359 |
| AcHexCmE (18:3) | -1.536361769 | 1.335395663 | TG (14:0e_6:0_11:1) | -3.33352273 | 1.313531419 |
| AcHexCmE (20:3) | -1.254446481 | 1.289382322 | TG (33:1e) | -1.822269396 | 1.298489498 |
| AcHexCmE (28:0) | 2.620287184 | 1.352800036 | TG (16:0e_6:0_11:4) | -3.002736832 | 1.303294115 |
| AcHexStE (18:3) | -1.985766981 | 1.357945471 | TG (33:6e) | -3.847638011 | 1.355278301 |
| AcHexZyE (19:1) | -1.374141235 | 1.296554174 | TG (14:1e_10:1_10:1) | -1.015299005 | 1.368242869 |
| AcHexZyE (19:2) | -1.53547918 | 1.335025947 | TG (34:4e) | -3.661767066 | 1.401951787 |
| AcHexZyE (19:3) | -3.053824866 | 1.354687694 | TG (18:2e_6:0_11:2) | -1.838424567 | 1.300599269 |
| AcHexZyE (21:2) | -1.254446481 | 1.289382322 | TG (20:0e_6:0_10:0) | -1.240113288 | 1.296989759 |
| Cer (d18:2_16:0+O) | -1.583036724 | 1.320134593 | TG (18:0e_6:0_12:1) | -0.592208445 | 1.291177908 |
| Cer (d18:2_16:1) | -1.715180709 | 1.316170802 | TG (6:0_12:1_18:1) | -1.096845574 | 1.299579515 |
| Cer (d18:0_24:0) | 1.309277865 | 1.344912555 | TG (12:0e_6:0_18:3) | -0.970348445 | 1.298509182 |
| Cer (d18:1_24:0+O) | -2.070026473 | 1.32930103 | TG (6:0_12:2_18:2) | -1.540645131 | 1.292378241 |
| Cer (d18:0_25:0) | 4.639572815 | 1.369545089 | TG (6:0_12:2_18:3) | -1.74056661 | 1.312283586 |
| Cer (d18:1_26:0+O) | -3.158867267 | 1.375455725 | TG (12:1e_6:0_18:4) | -1.669375514 | 1.385769255 |
| Cer (d18:2_26:0+O) | -1.715668625 | 1.314800537 | TG (14:1e_11:3_11:3) | -1.75634546 | 1.297135978 |
| Cer (t18:0_20:0) | -1.03039768 | 1.289901793 | TG (12:1e_10:3_14:3) | -3.960086828 | 1.372078467 |
| Cer (t18:0_20:0+O) | -0.972654107 | 1.307434167 | TG (38:2e) | -1.28292143 | 1.371091226 |
| Cer (t18:0_23:0) | 1.948923449 | 1.342183236 | TG (14:0e_6:0_18:3) | -2.146584016 | 1.343938991 |
| CerP (d37:1+O) | -2.632470917 | 1.33443706 | TG (4:0_14:2_20:3) | -2.171065003 | 1.359705587 |
| DG (6:0_12:2) | -3.259776034 | 1.377133871 | TG (38:5) | -1.590391839 | 1.323363515 |
| DG (18:4) | -5.098948162 | 1.338367281 | TG (38:6e) | -1.598863459 | 1.373651232 |
| DG (16:0_16:0) | -0.944589837 | 1.364999525 | TG (39:8e) | 0.922245332 | 1.387329332 |
| DG (34:3) | -1.425491144 | 1.303120802 | TG (20:1e_10:1_10:1) | -0.867852607 | 1.359805677 |
| DG (16:0_18:3) | -1.252759596 | 1.366473279 | TG (45:7) | -1.583311111 | 1.349145223 |
| DG (34:4) | -1.724250203 | 1.291940995 | TG (12:0_18:2_18:2) | -1.560930798 | 1.306105888 |
| DG (35:4) | -1.838424567 | 1.300599269 | TG (12:0_18:2_18:3) | -3.029791825 | 1.388318629 |
| DG (18:0_18:3) | -1.077797253 | 1.321851068 | TG (12:0_18:3_18:3) | -3.391615829 | 1.344897155 |
| DG (18:3_18:2) | -1.077330683 | 1.331515156 | TG (16:0_13:0_20:4) | -0.778910792 | 1.30734101 |
| DG (18:3_18:3) | -1.837505535 | 1.398375758 | TG (16:1_16:1_18:2) | -1.252512659 | 1.330616434 |
| DG (36:7) | -1.749103024 | 1.300143737 | TG (18:2_14:1_18:2) | -2.104224461 | 1.353176004 |
| DG (20:1_18:2) | -1.356827434 | 1.330369465 | TG (14:0_18:2_18:3) | -1.010619858 | 1.337428281 |
| DG (20:3_18:2) | -0.973623376 | 1.323204412 | TG (14:0_18:3_18:3) | -2.077229039 | 1.398921377 |
| DG (18:3_20:3) | -1.598863459 | 1.373651232 | TG (18:3_14:2_18:3) | -5.970177158 | 1.379811446 |
| DG (38:7e) | -1.754513736 | 1.313214348 | TG (18:3_14:3_18:3) | -5.304694479 | 1.368592931 |
| DG (40:4) | -0.936368409 | 1.30069785 | TG (29:0_6:0_16:1) | -0.635578712 | 1.390258876 |
| DG (26:0_18:3) | 0.885850074 | 1.293460611 | TG (15:0_18:2_18:2) | -1.096765498 | 1.334715611 |
| DGDG (16:0_16:0) | -1.558639854 | 1.317515814 | TG (15:0_18:2_18:3) | -1.113416535 | 1.302342699 |
| DGDG (16:0_18:1) | -2.17312234 | 1.33395659 | TG (15:0_18:3_18:3) | -1.504397749 | 1.376881525 |

| | | | | | |
|---|---|---|---|---|---|
| DGDG (16:0_18:2) | -1.602327923 | 1.319355115 | TG (18:4_15:0_18:3) | -2.241858639 | 1.397540836 |
| DGDG (18:1_18:1) | -2.80861706 | 1.337541807 | TG (18:3_11:2_22:4) | 0.590788216 | 1.305358509 |
| DGDG (18:1_18:2) | -1.975824287 | 1.358286667 | TG (16:0_18:1_18:3) | -1.728479893 | 1.361531891 |
| Hex1Cer (d18:2_16:0) | -1.315256576 | 1.306076599 | TG (16:1_18:3_18:3) | -1.907441079 | 1.39476335 |
| Hex1Cer (d18:2_16:1) | -1.614348977 | 1.332229174 | TG (18:4_16:1_18:3) | -3.118980205 | 1.400981566 |
| Hex1Cer (d18:1_20:0+O) | -1.252563108 | 1.345300722 | TG (52:8) | -5.744506123 | 1.370183977 |
| Hex1Cer (d18:2_22:5) | -2.485128915 | 1.350108951 | TG (18:3_14:3_20:3) | -2.415512526 | 1.348606407 |
| Hex1Cer (d18:2_24:1) | -1.070503192 | 1.309529209 | TG (52:9e) | -2.021770152 | 1.355830979 |
| Hex1Cer (d18:2_24:5) | -2.070026301 | 1.377390067 | TG (18:3_17:1_18:3) | -1.249547245 | 1.330512314 |
| Hex1Cer (d18:2_24:6) | -2.46917369 | 1.314808712 | TG (18:4_17:1_18:3) | -2.37020522 | 1.400202394 |
| Hex1Cer (t42:1+O) | -1.00177158 | 1.298329197 | TG (18:3_13:0_22:6) | -4.237650879 | 1.404128573 |
| MG (18:4) | -3.846915253 | 1.318707066 | TG (18:1_18:2_18:2) | -0.851818954 | 1.292331579 |
| MGDG (18:0_18:3) | -1.28066049 | 1.353886203 | TG (18:2_18:2_18:2) | -1.378477599 | 1.353755475 |
| MGDG (18:3_29:10) | -2.090716224 | 1.363397598 | TG (18:1_18:3_18:3) | -1.340262937 | 1.395085837 |
| MePC (31:0) | -2.140323496 | 1.358613277 | TG (54:7) | -4.555265518 | 1.364030212 |
| MePC (31:1) | -2.469085342 | 1.370824932 | TG (18:3_18:2_18:3) | -1.230436609 | 1.400340886 |
| MePC (32:0) | -1.640096882 | 1.313200046 | TG (18:3_18:3_18:3) | -1.604995379 | 1.396916954 |
| MePC (32:1) | -2.199380829 | 1.300267502 | TG (18:3e_18:3_18:3) | -6.998475267 | 1.394209369 |
| MePC (32:2) | -1.898057254 | 1.35352302 | TG (55:13) | -1.622279911 | 1.396431731 |
| MePC (33:0) | -1.773229948 | 1.360757747 | TG (19:1_18:2_18:2) | -1.968100049 | 1.363822257 |
| MePC (33:1) | -2.73225247 | 1.351859417 | TG (18:1_17:1_20:4) | -1.513769661 | 1.307047126 |
| MePC (33:2) | -2.624899242 | 1.391012973 | TG (19:1_18:3_18:3) | -1.519065619 | 1.359863601 |
| MePC (33:3) | -2.794573541 | 1.391036003 | TG (19:1_18:3_18:4) | -1.964652398 | 1.387491988 |
| MePC (34:2) | -2.600140362 | 1.379732261 | TG (20:0_16:0_20:4) | -2.329490907 | 1.31264815 |
| MePC (34:7) | -2.351811833 | 1.363534209 | TG (20:0_18:2_18:2) | -2.380975786 | 1.307648576 |
| MePC (35:0) | -1.718116644 | 1.342079089 | TG (20:1_18:3_18:3) | -2.452849555 | 1.378757026 |
| MePC (35:1) | -3.531823562 | 1.38134934 | TG (18:3_18:2_20:3) | -2.143376885 | 1.404866321 |
| MePC (35:3) | -3.055587517 | 1.392590997 | TG (18:3_18:3_20:2) | -1.904468183 | 1.397083499 |
| PA (15:0_18:2) | 3.073580524 | 1.348988585 | TG (18:4_14:3_24:2) | -2.239540663 | 1.319137292 |
| PA (15:0_18:3) | 1.38030769 | 1.38932937 | TG (18:3_18:3_20:3) | -1.378477599 | 1.353755475 |
| PC (10:0_18:2) | 1.34782767 | 1.385406594 | TG (20:1e_18:4_18:4) | -2.28462541 | 1.337252904 |
| PC (30:2) | 1.577150775 | 1.311420469 | TG (22:0_12:1_23:1) | -3.821258615 | 1.298701864 |
| PC (31:4) | -0.55187028 | 1.314827091 | TG (18:3_18:3_21:0) | -1.684721198 | 1.299139274 |
| PC (32:0) | -1.4510901 | 1.300721973 | TG (18:3_18:3_21:1) | -2.34134835 | 1.36748125 |
| PC (33:3) | -1.652678339 | 1.36559744 | TG (18:4_18:4_21:0) | -2.651673403 | 1.379758529 |
| PC (33:4) | -2.629791744 | 1.383760115 | TG (20:2e_18:4_20:4) | -2.041333331 | 1.363642551 |
| PC (16:0_18:1) | -1.860513479 | 1.337285063 | TG (20:3e_18:4_20:3) | -2.514997951 | 1.355257126 |
| PC (34:1) | -1.894343125 | 1.33820045 | TG (18:3e_20:4_20:4) | -2.226133381 | 1.376690751 |
| PC (34:3) | -1.924804997 | 1.313020368 | TG (18:4_18:4_22:4) | -2.239540663 | 1.319137292 |
| PC (34:4) | -2.317521317 | 1.347751836 | TG (18:1_18:2_22:0) | -1.46127584 | 1.304154196 |
| PC (34:5) | -1.498903035 | 1.363226615 | TG (18:1_18:3_22:0) | -1.868101683 | 1.324673624 |
| PC (35:5) | -1.883892389 | 1.370649014 | TG (22:0_18:2_18:3) | -1.943287988 | 1.379480256 |

| | | | | | |
|---|---|---|---|---|---|
| PC (36:1) | -1.601091014 | 1.298362457 | TG (22:0_18:3_18:3) | -2.394167921 | 1.389713415 |
| PC (18:1_18:1) | -2.100989943 | 1.33547551 | TG (20:1_18:1_20:4) | -1.063752616 | 1.314564111 |
| PC (36:3) | -1.804615624 | 1.35654015 | TG (18:3_18:3_22:1) | -2.95503735 | 1.385344954 |
| PC (19:0_18:3) | -1.412557736 | 1.358173524 | TG (18:4_18:4_22:1) | -3.095091698 | 1.343098687 |
| PC (37:5) | -2.716792807 | 1.382262452 | TG (18:3_18:3_22:3) | -3.000281432 | 1.352878116 |
| PC (38:3) | -1.718116644 | 1.342079089 | TG (20:1e_18:4_20:4) | -1.956738814 | 1.325919004 |
| PC (38:4) | -3.469813838 | 1.327185753 | TG (18:1_18:1_23:1) | -2.020030691 | 1.366649479 |
| PC (38:5) | -3.06315074 | 1.39543096 | TG (18:2_18:2_23:0) | -4.078579178 | 1.358640637 |
| PC (40:3) | -5.254207401 | 1.331603328 | TG (17:0_18:3_24:2) | -4.965476789 | 1.382994793 |
| PC (40:7) | -1.644833454 | 1.290545229 | TG (59:5) | -1.732253902 | 1.35642647 |
| PE (31:2) | 1.378045581 | 1.389694866 | TG (20:4e_20:4_20:4) | -1.948726554 | 1.326602217 |
| PE (18:1_18:1) | -1.50861311 | 1.321139617 | TG (60:13e) | -2.428258381 | 1.359586283 |
| PE (37:5) | -1.130947324 | 1.310954571 | TG (18:1_18:2_24:0) | -1.803599946 | 1.314531334 |
| PE (16:0_22:0) | -2.482283438 | 1.303288326 | TG (24:1_18:2_18:2) | -0.858254438 | 1.320208216 |
| PE (16:0_22:5) | -1.50861311 | 1.321139617 | TG (24:0_18:3_18:3) | -1.66293885 | 1.370815556 |
| PE (40:6) | -2.01814064 | 1.297105013 | TG (61:5) | -1.663971305 | 1.353589164 |
| PE (59:7) | 4.306554288 | 1.353596576 | WE (26:0_18:3) | -4.501247895 | 1.333410791 |
| PG (16:0_18:1) | -1.20895535 | 1.325394443 | WE (28:0_18:4) | -2.524059463 | 1.316492465 |
| PG (42:2) | -0.74218688 | 1.299828553 | ZyE (15:0) | -1.513558697 | 1.318712827 |
| PG (42:3) | -1.781280403 | 1.385376698 | Cer (d40:0) | 1.381209525 | 1.303476648 |
| PG (46:1) | -3.034328721 | 1.379627647 | Cer (t17:0_23:0) | 2.369851647 | 1.350953722 |
| PG (28:0_18:1) | -1.606342446 | 1.37919148 | Cer (t17:0_25:0) | 2.23505924 | 1.364963738 |
| PG (28:1_18:2) | -0.807703274 | 1.332591025 | Cer (t18:0_25:0) | 2.235442314 | 1.365314658 |
| PG (28:1_18:3) | -2.208879171 | 1.382229639 | DGDG (16:0_18:3) | -1.644532867 | 1.331048239 |
| PG (48:4) | -3.034328721 | 1.379627647 | DGDG (18:2_18:3) | -2.219935367 | 1.375883841 |
| PI (40:4) | -2.132271288 | 1.360916079 | LPC (18:3) | -2.917488934 | 1.374985391 |
| PI (42:4) | -1.569296786 | 1.323012941 | LPE (18:3) | -1.626601605 | 1.296691347 |
| PI (42:5) | -1.700708079 | 1.297519498 | MGDG (47:12) | -2.463932329 | 1.374036874 |
| PI (44:4) | -4.317575162 | 1.375510054 | PC (15:0_18:3) | -1.532076705 | 1.310707603 |
| PS (34:1) | -1.326948442 | 1.295971585 | PC (16:0_18:3) | -1.532076705 | 1.310707603 |
| SPH (d18:0) | -1.33584235 | 1.337314976 | PC (18:3_18:2) | -1.945477058 | 1.36534717 |
| SQDG (18:1_18:1) | -26.78070217 | 1.292181394 | PEt (38:6e) | 1.59097481 | 1.314812431 |
| SQDG (18:1_18:3) | -1.889021163 | 1.324885865 | | | |

**Table S3.** Shared DTLs of GF35 vs. GF30 and WF35 vs. WF30.

| Lipid name | GF35 vs. GF30 log2 (fold change) | WF35 vs. WF30 log2 (fold change) | Lipid name | GF35 vs. GF30 log2 (fold change) | WF35 vs. WF30 log2 (fold change) |
|---|---|---|---|---|---|
| AcHexCmE (18:2) | 1.785615993 | -1.375817716 | PG (28:0_18:1) | 1.290725208 | -1.606342446 |
| AcHexStE (18:3) | 1.294712311 | -1.985766981 | PI (42:5) | 1.036789596 | -1.700708079 |
| AcHexZyE (19:1) | 1.785906063 | -1.374141235 | SQDG (18:1_18:1) | 1.769392225 | -26.78070217 |
| DG (20:1_18:2) | 1.555148927 | -1.356827434 | TG (18:1_18:1_23:1) | 1.434685511 | -2.020030691 |
| DGDG (18:1_18:1) | 2.684110335 | -2.80861706 | TG (18:1_18:2_18:2) | 0.926462018 | -0.851818954 |
| DGDG (18:2_18:3) | 1.038156213 | -2.219935367 | TG (18:1_18:2_24:0) | 1.882359295 | -1.803599946 |
| Hex1Cer (d18:2_22:5) | -3.33887471 | -2.485128915 | TG (18:3_18:3_21:1) | -1.428130179 | -2.34134835 |
| Hex1Cer (d18:2_24:1) | -0.848278825 | -1.070503192 | TG (18:3e_18:3_18:3) | 1.399180054 | -6.998475267 |
| Hex1Cer (d18:2_24:5) | -2.689054856 | -2.070026301 | TG (20:1_18:3_18:3) | 1.300615312 | -2.452849555 |
| MePC (32:2) | -3.805227939 | -1.898057254 | TG (24:0_18:3_18:3) | 1.503262746 | -1.66293885 |
| MePC (34:2) | 1.314596961 | -2.600140362 | TG (24:1_18:2_18:2) | 1.411340081 | -0.858254438 |
| MePC (35:1) | 2.265084044 | -3.531823562 | TG (38:2e) | 2.86669018 | -1.28292143 |
| PC (16:0_18:1) | 1.195313934 | -1.860513479 | TG (38:5) | 3.11292087 | -1.590391839 |
| PC (18:1_18:1) | 2.157580428 | -2.100989943 | TG (52:9e) | 1.348675018 | -2.021770152 |
| PC (38:4) | 2.269306842 | -3.469813838 | TG (6:0_12:1_18:1) | 3.113871718 | -1.096845574 |

**Table S4.** Specific DTLs in GF35 vs. GF30.

| Lipid name | log2 (fold change) | Lipid name | log2 (fold change) | Lipid name | log2 (fold change) |
|---|---|---|---|---|---|
| PS (16:0_18:3) | 24.44397528 | AcHexZyE (18:1) | 2.240610353 | PA (18:1_18:2) | 1.389193722 |
| PC (60:1) | 4.554500477 | CmE (26:6) | 2.132279882 | PE (34:2) | 1.389193722 |
| DG (36:1) | 4.195572129 | AcHexZyE (19:0) | 2.111697294 | ZyE (21:4) | 1.341482067 |
| TG (6:0_18:3_18:3) | 3.76078143 | AcHexCmE (18:1) | 2.106338679 | PC (33:2) | 1.292993922 |
| TG (18:1_18:1_24:0) | 3.53817035 | AcHexSiE (17:1) | 2.106338679 | PE (18:0_18:2) | 1.279176752 |
| TG (6:0_12:0_18:1) | 3.486609184 | PC (19:1_18:1) | 2.105400699 | DG (36:3e) | 1.251644428 |
| PC (20:1_13:0) | 3.379417819 | TG (30:0_6:0_6:0) | 2.089087858 | DG (38:6e) | 1.251644428 |
| PE (36:1) | 3.379417819 | TG (18:4_14:3_24:0) | 2.014092512 | MG (22:1) | 1.247416759 |
| PG (46:2) | 3.130810233 | PE (36:2) | 2.012488261 | PA (36:3) | 1.201807091 |
| PA (20:1_18:3) | 3.118814674 | TG (20:1_18:2_18:2) | 1.987238332 | PG (31:0) | 1.15972898 |
| AcHexSiE (20:1) | 3.089213989 | AcHexChE (18:3) | 1.973972434 | DG (32:2e) | 1.071705863 |
| AcHexStE (20:0) | 3.089213989 | TG (24:1_14:4_18:3) | 1.846227807 | DG (34:5e) | 1.064437216 |
| LPE (20:1) | 3.062762427 | TG (45:8e) | 1.826999985 | DGDG (18:1_18:3) | 1.035624905 |
| PE (20:1e) | 3.032696692 | PC (18:1_18:2) | 1.826848076 | TG (16:1e_18:3_18:3) | 1.010648953 |
| ChE (23:5) | 2.960747644 | TG (20:3e_18:1_18:4) | 1.816291096 | AcHexStE (20:1) | 0.978030687 |
| ZyE (23:4) | 2.960091338 | PC (18:1_18:3) | 1.799283165 | TG (54:10e) | 0.93512999 |
| CmE (28:6) | 2.938231699 | DG (34:1e) | 1.790203226 | AcHexStE (18:1) | 0.918542538 |
| SQDG (47:7) | 2.934827468 | PG (28:1_18:1) | 1.739006786 | AcHexSiE (18:2) | 0.915980281 |
| AcHexChE (18:1) | 2.929293746 | PA (18:0_18:2) | 1.727510812 | DGDG (18:3_20:3) | 0.73901626 |
| PC (18:1_13:0) | 2.923978171 | PE (16:0_18:1) | 1.727510812 | TG (16:1_6:0_22:0) | 0.673076547 |
| PG (34:2) | 2.902785868 | AcHexChE (26:0) | 1.672123195 | SPH (t20:0) | 0.548792425 |
| AcHexCmE (20:1) | 2.772845796 | PG (34:4) | 1.633404345 | Cer (d34:0) | 0.429374876 |
| DG (20:1_18:1) | 2.592117239 | AcHexSiE (24:0) | 1.625337497 | TG (18:0_18:0_19:0) | 0.195486842 |
| PEt (18:1_18:2) | 2.587103324 | PE (16:0_20:1) | 1.587232297 | CmE (32:2) | -0.246981341 |
| PA (20:1_18:2) | 2.561542727 | PC (41:1e) | 1.572289816 | ZyE (33:1) | -0.246981341 |
| AcHexCmE (20:2) | 2.513425274 | PE (18:0_16:0) | 1.553400106 | Cer (d28:0) | -0.838639384 |
| MGDG (18:1_18:2) | 2.421385835 | PE (18:0_18:3) | 1.553400106 | TG (56:3) | -0.841021517 |
| TG (16:0e_6:0_22:1) | 2.404737792 | MG (24:1) | 1.526718316 | TG (18:3_14:1_18:3) | -1.069300666 |
| DG (18:1_18:1) | 2.379611695 | TG (18:1_18:1_18:2) | 1.515571037 | cPA (16:0) | -1.303898228 |
| MGDG (18:1_18:1) | 2.35444369 | TG (22:3_12:4_22:3) | 1.500244209 | Hex1Cer (t42:2) | -1.481763662 |
| TG (12:0e_10:0_22:1) | 2.332368662 | TG (52:6e) | 1.439230325 | LPMe (16:0) | -1.962904773 |
| TG (41:8e) | 2.322743681 | AcHexStE (18:0) | 1.435874558 | PI (16:0_16:0) | -2.392720567 |
| PA (20:1_18:1) | 2.307714965 | AcHexSiE (18:1) | 1.435231092 | PC (18:0_14:2) | -4.133017738 |
| AcHexChE (18:2) | 2.306939018 | AcHexZyE (22:2) | 1.432567407 | BisMePA (17:0_18:3) | -4.135506018 |
| TG (41:10) | 2.29948597 | AcHexSiE (20:3) | 1.4325449 | PMe (18:0_18:3) | -4.555008203 |
| AcHexSiE (19:1) | 2.267477082 | AcHexStE (20:2) | 1.43198254 | | |
| TG (18:1_20:3_22:0) | 2.255089561 | DG (32:1e) | 1.393135096 | | |

**Table S5.** Specific DTLs in WF35 vs. WF30.

| Lipid name | log2 (fold change) | Lipid name | log2 (fold change) | Lipid name | log2 (fold change) |
|---|---|---|---|---|---|
| Cer (d18:0_25:0) | 4.639572815 | TG (19:1_18:3_18:3) | -1.519065619 | MePC (31:0) | -2.140323496 |
| PE (59:7) | 4.306554288 | PC (15:0_18:3) | -1.532076705 | TG (18:3_18:2_20:3) | -2.143376885 |
| PA (15:0_18:2) | 3.073580524 | PC (16:0_18:3) | -1.532076705 | TG (14:0e_6:0_18:3) | -2.146584016 |
| AcHexCmE (28:0) | 2.620287184 | AcHexZyE (19:2) | -1.53547918 | TG (4:0_14:2_20:3) | -2.171065003 |
| Cer (t17:0_23:0) | 2.369851647 | AcHexCmE (18:3) | -1.536361769 | DGDG (16:0_18:1) | -2.17312234 |
| Cer (t18:0_25:0) | 2.235442314 | TG (6:0_12:2_18:2) | -1.540645131 | MePC (32:1) | -2.199380829 |
| Cer (t17:0_25:0) | 2.23505924 | DGDG (16:0_16:0) | -1.558639854 | PG (28:1_18:3) | -2.208879171 |
| Cer (t18:0_23:0) | 1.948923449 | TG (12:0_18:2_18:2) | -1.560930798 | TG (18:3e_20:4_20:4) | -2.226133381 |
| PEt (38:6e) | 1.59097481 | PI (42:4) | -1.569296786 | TG (18:4_14:3_24:2) | -2.239540663 |
| PC (30:2) | 1.577150775 | Cer (d18:2_16:0+O) | -1.583036724 | TG (18:4_18:4_22:4) | -2.239540663 |
| Cer (d40:0) | 1.381209525 | TG (45:7) | -1.583311111 | TG (18:4_15:0_18:3) | -2.241858639 |
| PA (15:0_18:3) | 1.38030769 | DG (18:3_20:3) | -1.598863459 | TG (20:1e_18:4_18:4) | -2.28462541 |
| PE (31:2) | 1.378045581 | TG (38:6e) | -1.598863459 | PC (34:4) | -2.317521317 |
| PC (10:0_18:2) | 1.34782767 | PC (36:1) | -1.601091014 | TG (20:0_16:0_20:4) | -2.329490907 |
| Cer (d18:0_24:0) | 1.309277865 | DGDG (16:0_18:2) | -1.602327923 | MePC (34:7) | -2.351811833 |
| TG (39:8e) | 0.922245332 | TG (18:3_18:3_18:3) | -1.604995379 | TG (18:4_17:1_18:3) | -2.37020522 |
| DG (26:0_18:3) | 0.885850074 | Hex1Cer (d18:2_16:1) | -1.614348977 | TG (20:0_18:2_18:2) | -2.380975786 |
| TG (18:3_11:2_22:4) | 0.590788216 | TG (55:13) | -1.622279911 | TG (22:0_18:3_18:3) | -2.394167921 |
| PC (31:4) | -0.55187028 | LPE (18:3) | -1.626601605 | TG (18:3_14:3_20:3) | -2.415512526 |
| TG (18:0e_6:0_12:1) | -0.592208445 | MePC (32:0) | -1.640096882 | TG (60:13e) | -2.428258381 |
| TG (29:0_6:0_16:1) | -0.635578712 | DGDG (16:0_18:3) | -1.644532867 | MGDG (47:12) | -2.463932329 |
| PG (42:2) | -0.74218688 | PC (40:7) | -1.644833454 | MePC (31:1) | -2.469085342 |
| TG (16:0_13:0_20:4) | -0.778910792 | PC (33:3) | -1.652678339 | Hex1Cer (d18:2_24:6) | -2.46917369 |
| PG (28:1_18:2) | -0.807703274 | TG (61:5) | -1.663971305 | PE (16:0_22:0) | -2.482283438 |
| TG (20:1e_10:1_10:1) | -0.867852607 | TG (12:1e_6:0_18:4) | -1.669375514 | TG (20:3e_18:4_20:3) | -2.514997951 |
| DG (40:4) | -0.936368409 | TG (18:3_18:3_21:0) | -1.684721198 | WE (28:0_18:4) | -2.524059463 |
| DG (16:0_16:0) | -0.944589837 | Cer (d18:2_16:1) | -1.715180709 | MePC (33:2) | -2.624899242 |
| TG (12:0e_6:0_18:3) | -0.970348445 | Cer (d18:2_26:0+O) | -1.715668625 | PC (33:4) | -2.629791744 |
| Cer (t18:0_20:0+O) | -0.972654107 | MePC (35:0) | -1.718116644 | CerP (d37:1+O) | -2.632470917 |
| DG (20:3_18:2) | -0.973623376 | PC (38:3) | -1.718116644 | TG (18:4_18:4_21:0) | -2.651673403 |
| Hex1Cer (t42:1+O) | -1.00177158 | DG (34:4) | -1.724250203 | PC (37:5) | -2.716792807 |
| TG (14:0_18:2_18:3) | -1.010619858 | TG (16:0_18:1_18:3) | -1.728479893 | MePC (33:1) | -2.73225247 |
| TG (14:1e_10:1_10:1) | -1.015299005 | TG (59:5) | -1.732253902 | MePC (33:3) | -2.794573541 |
| Cer (t18:0_20:0) | -1.03039768 | TG (6:0_12:2_18:3) | -1.74056661 | LPC (18:3) | -2.917488934 |
| TG (20:1_18:1_20:4) | -1.063752616 | DG (36:7) | -1.749103024 | TG (18:3_18:3_22:1) | -2.95503735 |
| DG (18:3_18:2) | -1.077330683 | DG (38:7e) | -1.754513736 | TG (18:3_18:3_22:3) | -3.000281432 |
| DG (18:0_18:3) | -1.077797253 | TG (14:1e_11:3_11:3) | -1.75634546 | TG (16:0e_6:0_11:4) | -3.002736832 |
| TG (15:0_18:2_18:2) | -1.096765498 | MePC (33:0) | -1.773229948 | TG (12:0_18:2_18:3) | -3.029791825 |
| TG (15:0_18:2_18:3) | -1.113416535 | PG (42:3) | -1.781280403 | PG (46:1) | -3.034328721 |

| | | | | | |
|---|---|---|---|---|---|
| PE (37:5) | -1.130947324 | PC (36:3) | -1.804615624 | PG (48:4) | -3.034328721 |
| PG (16:0_18:1) | -1.20895535 | TG (33:1e) | -1.822269396 | AcHexZyE (19:3) | -3.053824866 |
| TG (18:3_18:2_18:3) | -1.230436609 | DG (18:3_18:3) | -1.837505535 | MePC (35:3) | -3.055587517 |
| TG (20:0e_6:0_10:0) | -1.240113288 | DG (35:4) | -1.838424567 | PC (38:5) | -3.06315074 |
| TG (18:3_17:1_18:3) | -1.249547245 | TG (18:2e_6:0_11:2) | -1.838424567 | TG (18:4_18:4_22:1) | -3.095091698 |
| TG (16:1_16:1_18:2) | -1.252512659 | TG (18:1_18:3_22:0) | -1.868101683 | TG (18:4_16:1_18:3) | -3.118980205 |
| Hex1Cer (d18:1_20:0+O) | -1.252563108 | PC (35:5) | -1.883892389 | Cer (d18:1_26:0+O) | -3.158867267 |
| DG (16:0_18:3) | -1.252759596 | SQDG (18:1_18:3) | -1.889021163 | DG (6:0_12:2) | -3.259776034 |
| AcHexCmE (20:3) | -1.254446481 | PC (34:1) | -1.894343125 | TG (14:0e_6:0_11:1) | -3.33352273 |
| AcHexZyE (21:2) | -1.254446481 | TG (18:3_18:3_20:2) | -1.904468183 | TG (12:0_18:3_18:3) | -3.391615829 |
| MGDG (18:0_18:3) | -1.28066049 | TG (16:1_18:3_18:3) | -1.907441079 | TG (34:4e) | -3.661767066 |
| Hex1Cer (d18:2_16:0) | -1.315256576 | PC (34:3) | -1.924804997 | TG (22:0_12:1_23:1) | -3.821258615 |
| PS (34:1) | -1.326948442 | TG (22:0_18:2_18:3) | -1.943287988 | MG (18:4) | -3.846915253 |
| SPH (d18:0) | -1.33584235 | PC (18:3_18:2) | -1.945477058 | TG (33:6e) | -3.847638011 |
| TG (18:1_18:3_18:3) | -1.340262937 | TG (20:4e_20:4_20:4) | -1.948726554 | TG (12:1e_10:3_14:3) | -3.960086828 |
| TG (18:2_18:2_18:2) | -1.378477599 | TG (20:1e_18:4_20:4) | -1.956738814 | TG (18:2_18:2_23:0) | -4.078579178 |
| TG (18:3_18:3_20:3) | -1.378477599 | TG (19:1_18:3_18:4) | -1.964652398 | TG (18:3_13:0_22:6) | -4.237650879 |
| PC (19:0_18:3) | -1.412557736 | TG (19:1_18:2_18:2) | -1.968100049 | PI (44:4) | -4.317575162 |
| DG (34:3) | -1.425491144 | DGDG (18:1_18:2) | -1.975824287 | WE (26:0_18:3) | -4.501247895 |
| PC (32:0) | -1.4510901 | PE (40:6) | -2.01814064 | TG (54:7) | -4.555265518 |
| TG (18:1_18:2_22:0) | -1.46127584 | TG (20:2e_18:4_20:4) | -2.041333331 | TG (17:0_18:3_24:2) | -4.965476789 |
| PC (34:5) | -1.498903035 | Cer (d18:1_24:0+O) | -2.070026473 | DG (18:4) | -5.098948162 |
| TG (15:0_18:3_18:3) | -1.504397749 | TG (14:0_18:3_18:3) | -2.077229039 | PC (40:3) | -5.254207401 |
| PE (16:0_22:5) | -1.50861311 | MGDG (18:3_29:10) | -2.090716224 | TG (18:3_14:3_18:3) | -5.304694479 |
| PE (18:1_18:1) | -1.50861311 | TG (18:2_14:1_18:2) | -2.104224461 | TG (52:8) | -5.744506123 |
| ZyE (15:0) | -1.513558697 | PI (40:4) | -2.132271288 | TG (18:3_14:2_18:3) | -5.970177158 |
| TG (18:1_17:1_20:4) | -1.513769661 | ST (t18:1_24:0+O) | -2.139959266 | | |

**Table S6.** Statistics of lipid species in GF35 vs. GF30 and WF35 vs. WF30.

| lipid name | DELs number in GF35 vs. GF30 | proportion in GF35 vs. GF30 | lipid name | DELs number in WF35 vs. WF30 | proportion in WF35 vs. WF30 |
|---|---|---|---|---|---|
| TG | 35 | 25.2% | TG | 96 | 42.3% |
| PC | 12 | 8.6% | PC | 25 | 11.0% |
| DG | 10 | 7.2% | DG | 17 | 7.5% |
| PE | 9 | 6.5% | Cer | 15 | 6.6% |
| AcHexSiE | 7 | 5.0% | MePC | 14 | 6.2% |
| AcHexStE | 6 | 4.3% | Hex1Cer | 8 | 3.5% |
| PA | 6 | 4.3% | PG | 8 | 3.5% |
| PG | 6 | 4.3% | DGDG | 7 | 3.1% |
| AcHexChE | 4 | 2.9% | PE | 7 | 3.1% |
| AcHexCmE | 4 | 2.9% | AcHexCmE | 4 | 1.8% |
| AcHexZyE | 4 | 2.9% | AcHexZyE | 4 | 1.8% |
| DGDG | 4 | 2.9% | PI | 4 | 1.8% |
| Hex1Cer | 4 | 2.9% | MGDG | 3 | 1.3% |
| CmE | 3 | 2.2% | PA | 2 | 0.9% |
| MePC | 3 | 2.2% | SQDG | 2 | 0.9% |
| ZyE | 3 | 2.2% | WE | 2 | 0.9% |
| Cer | 2 | 1.4% | AcHexStE | 1 | 0.4% |
| MG | 2 | 1.4% | MG | 1 | 0.4% |
| MGDG | 2 | 1.4% | PS | 1 | 0.4% |
| PI | 2 | 1.4% | SPH | 1 | 0.4% |
| SQDG | 2 | 1.4% | ST | 1 | 0.4% |
| BisMePA | 1 | 0.7% | ZyE | 1 | 0.4% |
| ChE | 1 | 0.7% | LPC | 1 | 0.4% |
| LPE | 1 | 0.7% | LPE | 1 | 0.4% |
| PS | 1 | 0.7% | PEt | 1 | 0.4% |
| SPH | 1 | 0.7% | | | |
| LPMe | 1 | 0.7% | | | |
| PEt | 1 | 0.7% | | | |
| PMe | 1 | 0.7% | | | |
| cPA | 1 | 0.7% | | | |
